# Inhibitory effect of Astragalus polysaccharide on osteoporosis in ovariectomized rats by regulating FoxO3a /Wnt signaling pathway^1^

**DOI:** 10.1590/s0102-865020190050000002

**Published:** 2019-06-03

**Authors:** Li Ou, Peifeng Wei, Min Li, Feng Gao

**Affiliations:** I PhD, Associate Professor, Department of Clinical Chinese Pharmacy, College of Pharmacy, Shaanxi University of Chinese Medicine, Xianyang, China. Design of the study, critical revision, final approval.; II PhD, Full Professor, Department of Clinical Chinese Pharmacy, College of Pharmacy, Shaanxi University of Chinese Medicine, Xianyang, China. Design of the study, acquisition of data, statistical analysis, critical revision, final approval.

**Keywords:** Astragalus Plant, Osteoporosis, Ovariectomy, Rats

## Abstract

**Purpose:**

To investigate inhibitory effect of Astragalus polysaccharide (APS) on osteoporosis in ovariectomized rats by regulating FoxO3a/Wnt2 signaling pathway.

**Methods:**

Postmenopausal osteoporosis (PMOP) animal model was developed by excising the bilateral ovaries of rats. The model rats were administered with APS (200 mg/kg, 400 mg/kg, 800 mg/kg) by intragastric administration once daily for 12 weeks. Bone density, bone metabolism index and oxidative stress index were measured in all groups. Furthermore, the regulation of APS of FoxO3a / Wnt2 signaling pathway was observed.

**Results:**

APS has an estrogen-like effect, which can increase bone mass, lower serum ALP and BGP values, increase blood calcium content, and increase bone density of the femur and vertebrae in rats. At the same time, APS can increase the bone mineral content of the femur, increase the maximum stress, maximum load and elastic modulus of the ovariectomized rats, improve oxidative stress in rats by increasing the gene expression of β-catenin and Wnt2 mRNA and inhibiting the gene expression of FoxO3a mRNA.

**Conclusion:**

Astragalus polysaccharide can effectively alleviate oxidative stress-mediated osteoporosis in ovariectomized rats, which may be related to its regulation of FoxO3a/Wnt2/β-catenin pathway.

## Introduction

 As the global population ages, the incidence of postmenopausal osteoporosis (PMOP) is increasing^1^. It is mainly characterized by bone mass reduction and micro structural degeneration of bone tissue, which affects the health of middle-aged and elderly women and reduces their quality of life^2^ ^,^ ^3^. However, the current pathogenesis of PMOP is not yet clear. In recent years, oxidative stress has been highly valued as a risk factor for PMOP. More and more studies have shown that under the stimuli of aging or estrogen levels reduction, a large accumulation of ROS in the body induces oxidative stress, which causes osteoporosis by activating FoxO3a and inhibiting the Wnt signaling pathway.

 A large number of reports from clinical trials have suggested that estrogen replacement therapy is useful in the management of PMOP. However, long-term and extensive use of estrogen increases the risk of cancers such as breast cancer and endometrial cancer^4^ ^-^ ^6^. Radix Astragali, the dried root of *Astragalus membranaceus* (Fisch.) Bunge, is one of the commonly used Chinese medicinal herbs in Asia region, and has a long history of clinical application. Astragalus polysaccharide (APS) is a major active ingredient of Astragalus membranaceus, which can alleviate the symptoms of PMOP, but the mechanism of treatment is still unclear^7^ ^-^ ^9^. 

 In this paper, PMOP animal model was established by excising the bilateral ovaries of rats to study the effects of APS on bone mineral density (BMD), osseous pathologic change, bone metabolism index and oxidative stress index in ovariectomized rats, and to explore its regulation mechanism on FoxO3a/Wnt signaling pathway.

## Methods

### Drug

 The purity of APS is more than 95%, which was purchased from Nanjing Jingzhu Biological Technology Co., Ltd. FOXO3a, Wnt2, LRP5 and β-catenin primers were designed using Primer Design software and provided by GenScript Biotechnology Co., Ltd. TransStart Tip Green qRCR SuperMix, TransScript All-in-One First-Strand cDNA Synthesis SuperMix for Qpcr (One-Step gDNA Removal), EasyPureTM RNA Kit were provided by Beijing Quanjin Biotechnology Co., Ltd. 

### Rat model of PMOP

 Six-month old SD rats were purchased from Experimental Animal Center of Fourth Military Medical University. All rat were maintained in specific pathogen-free (SPF) conditions and the operation of animal experiment conformed to the Animal Care and Use Committee of Shaanxi University of Chinese Medicine. All animals were housed in polypropilene cages at least 7 days before the beginning of the study, under a well ventilated environment, the humidity was 50% - 60%. During the experiment, the animals were kept in polypropylene cages at 20-24°C room temperature and controlled periods of light/dark (12/12 hours), receiving free access to water and food. The weight and feed consumption were monitored three times per week until the end of the experiment.

 The PMOP model was established by excising the bilateral ovaries of the rats^10^.After the operation, the rats were intramuscularly injected with 400,000 U/kg of penicillin once a day for 3 days. On the 7th day after the operation, exfoliated vaginal epithelial cells were examined by smear test and continuously examined for 5 days. There were no keratinized epithelial cells in the vaginal smear as an indicator of success in the rat model of PMOP. During the modeling period, no rat died.

### Animal grouping and treatment

 Sixty SD rats were randomly divided into sham operation group, model group, positive control group (Nilestriol 0.18 mg/kg dose, administered once every 2 weeks), and low-, middle- and high-dose APS group (APS 200mg/kg, 400mg/kg, 800mg/kg dose, administered once a day), 10 rats in each group. Except for the sham operation group, the 5 other groups of rats were resected bilateral ovaries. The sham operation and model groups were injected with equal amount of physiological saline. 

### Index of bone metabolism and oxidative stress in serum assay

 After 12 weeks of administration, the animals in each group were weighed, anesthetized with 3% sodium pentobarbital at 1 ml/kg, and blood was taken from the celiac artery to measure the content of 17β-estradiol (E_2_), calcium (Ca), alkaline phosphatase (ALP), osteocalcin (OC), glutathione peroxidase (GSH-Px), superoxide dismutase(SOD), malondialdehyde(MDA) in serum. 

### Bone biomechanical index assay and histologic examination

 After the rats were killed, the right femur was taken, the attached muscle tissue was peeled off, and the BMD values of the right femur and L_3_ vertebral bodies of the rats were examined by dual-energy X-ray bone densitometer (Discovery Wi, Hologic, USA). The three-point bending test of the whole bone was performed with a universal electronic material testing machine(CSS-44100, Changchun, China). Instrument parameters: loading speed 6 mm/min, maximum load 200 N, span 20 mm, trace load-deformation curve, calculate maximum stress, structural load and elastic modulus. The femur was fixed with 10% formalin for 24 hours, and then decalcified with EDTA for 60 days. After decalcification, a 0.3cm long femur was taken at the end of the fracture. The remaining femur was made into a longitudinal section along the middle of the femoral head. Both bone tissues were routinely paraffin-embedded. After HE staining, histologic examination was performed under light microscope.

### Real-time quantitative PCR detection of FOXO3a, Wnt2, LRP5 and β-catenin mRNA expression

 The left femur of the rat was taken and the attached muscle tissue was peeled off. The femur was cut, placed in a mortar filled with liquid nitrogen, and the corresponding volume of Trizol was added to extract RNA. Reverse transcription was carried out according to the kit instructions, pre-denaturation at 94℃ for 4 min on a PCR machine, denaturation at 94℃ for 30 sec, annealing at 31℃ for 30 sec, and extension at 72℃ for 31 sec for 40 cycles. The threshold value of the target gene was compared with the internal reference, and the relative expression amount was calculated.

### PCR primer synthesis

 FOXO3a, Wnt2, LRP5 and β-catenin primers were designed using Primer Design software and provided by GenScript Biotechnology Co., Ltd. The sequence is shown in Table 1.

**Table 1:** Primers used for qRT-PCR.

Gene	Forward primers (5′～3′)	Reverse primers (5′～3′)	Primer length
FOXO3a	GCACCAATTCTAACGCCAGCAC	ATCCAGCAGGTCGTCCATGAG G	240 bp
Wnt2	GGCCTTTGTTTACGCCATCTC	TCCTTCCAGCTCTGTTGTTG	127bp
LRP5	GACATTTACTGGCCCAATGG	CTGCCCTCCACCACCTTCT	131bp
β-Catenin	GAATGTCTGAGGACAAGCCACAAG	TGGGCACCAATATCAAGTCCAA	127bp
β-actin catenin	GGA AAG CAA GCT CAT CAT TCT	GGA AAG CAA GCT CAT CAT TCT	171bp

### Statistical analysis

 The data was statistically processed using SPSS 19.0 software. Measurement data were expressed as mean ± standard deviation (±s), and comparison between groups was analyzed by one-way analysis of variance. Significance was accepted at a level of p<0.05.

## Results

### Effect on indexes of bone metabolism in the serum of rats

 Compared with sham operation group, the E_2_ in the serum of the model group was significantly decreased (p<0.01), the OC and ALP levels were significantly increased (P<0.01), and the value of serum Ca was significantly decreased (p<0.05). Compared with model group, E_2_ level in the APS group (200mg/kg, 400mg/kg, 800mg/kg dose) and nylestriol group increased significantly (P<0.05), OC and ALP levels decreased to some extent, and ALP and OC values in the high-dose APS group (800mg/kg dose) decrease significantly (p<0.01). The content of Ca of each drug-administered group increased to a certain extent, and the APS group (400mg/kg, 800mg/kg dose) increased significantly, and the difference was statistically significant (p<0.05, Table 2). 

**Table 2:** Effect on index of bone metabolism in the serum of rats (±s, n=10).

Group	E_2_ (ng/L)	ALP(U/L)	OC(μg/L)	Ca(mmol/L)
Sham	46.23±11.07	85.74±18.55	2.34±0.52	2.28±0.45
model	26.54±11.25^a^	127.82±30.12^a^	3.12±0.39^a^	1.76±0.38^b^
Nylestriol	41.07±9.72^c^	98.39±23.64^d^	2.62±0.34^c^	2.14±0.29^d^
Low-dose APS	36.65±10.14^d^	103.48±20.36^d^	2.71±0.45^d^	1.97±0.25
Middle-dose APS	38.58±8.60^d^	95.26±29.37^d^	2.65±0.58^d^	2.08±0.20^d^
High-dose APS	38.95±12.39^d^	96.53±15.81^c^	2.66±0.27^c^	2.09±0.31^d^

^a^ p<0.01, ^b^ p<0.05, compared with sham group; ^c^ p <0.01, ^d^ p <0.05, compared with model group.

### Effects on BMD of the femur and vertebral in rats

**Figure 1:**
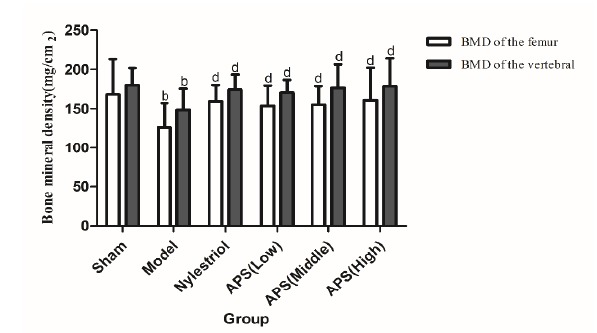
Effects on BMD of the femur and vertebral in rats. ^b^ P<0.05 compared with sham group; ^d^ P<0.05 compared with model group.

 Compared with sham operation group, BMD of the femur and vertebral in rats of the model control group decreased significantly (p<0.05). However, compared with model control group, BMD of the femur and vertebral of the rats in each administration group increased significantly. BMD of the femur and vertebrae was significantly increased in the APS group (200mg/kg, 400mg/kg, 800mg/kg dose, p<0.05). And the increase of BMD was positively correlated with the dose (Fig. 1). 

### Effects on bone biomechanics in rats

 Compared with sham operation group, the maximum stress, maximum load and elastic modulus of the femurs of the ovariectomized rats in the model control group were significantly decreased (p<0.05). After administration, the maximum load of the femurs in each dose group of APS was significantly better than that of model control group (p<0.01), the maximum stress and elastic modulus of the femurs in the middle- and high-dose groups of APS (400mg/kg, 800mg/kg dos) were significantly different from those in model control group (p<0.05, Table 3).

**Table 3:** Effects on bone biomechanics after treatment in different groups (±s, n=10).

Group	Maximum stress(mpa)	Maximum load(n)	Elastic modulus (mpa)
Sham	9.85±1.78	191.63±38.72	125.78±34.50
model	7.30±1.45^a^	140.27±25.64^a^	92.41±23.69^b^
Nylestriol	8.74±1.29^d^	185.23±30.51^c^	118.38±28.24^d^
Low-dose APS	8.66±1.42^d^	179.89±35.06^c^	116.23±25.91^d^
Middle-dose APS	8.66±1.36^d^	184.65±41.37^c^	117.92±21.33^d^
High-dose APS	8.70±1.51^d^	186.74±26.96^c^	119.25±28.17^d^

^a^ P<0.01 compared with sham group; ^b^ P<0.05 compared with sham group; ^c^ P<0.01 compared with model group; ^d^ P<0.05 compared with model group.

### Effect on the osseous pathologic changes of rats

**Figure 2:**
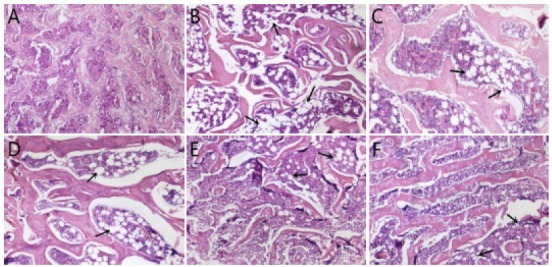
Effect on the osseous pathologic changes of rats (HE×40). (**A**) Sham operation group. (**B**) Model group. (**C**) Nilestriol group. (**D**) Low-dose APS group. (**E**) Middle-dose APS group. (**F**) High-dose APS group.

 As shown by histopathological examination results, the bone trabecular in the cancellous bone of the sham operation group was arranged regularly and densely, and there were many hematopoietic cells in the medullary cavity, but no fat cell hyperplasia and hypertrophy were observed (Fig. 2A). In the model group, substantia compacta was generally thinner, the number of trabecular bone was reduced, and the trabecular space was widened and sparse. The number of hematopoietic cells in the medullary cavity was reduced and the fat cells were densely packed, indicating that the osteoporosis model was made successfully (Fig. 2B). Compared with model group, the number of trabecular bone in each dose group of APS and Nylestriol group increased to different degrees, the trabecular space was narrowed, the number of hematopoietic cells in the medullary cavity increased, and the number of fat cells decreased, among which the number of trabecular bone of high dose APS group (800mg/kg dose) increased most significantly (Fig. 2C-F).

### Effect on oxidative stress index in rats

**Figure 3:**
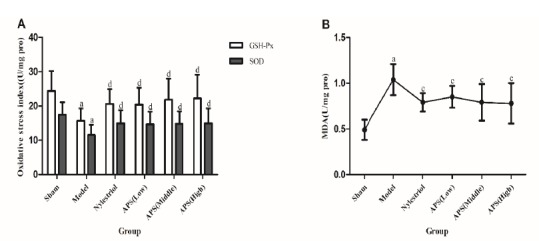
Effect on oxidative stress index in rats in each group. (**A**) Effect on GSH-Px and SOD levels in the serum of rats. (**B**) Effect on MDA level in the serum of rats. ^a^ P<0.01 compared with sham group; ^c^ P<0.01 compared with model group; ^d^ P<0.05 compared with model group.

 Compared with sham operation group, the GSH-Px and SOD levels in the femur of rats in the model group were significantly decreased (p<0.01), and the MDA level was significantly increased (p<0.01). Compared with model group, the level of MDA in the APS groups (200mg/kg, 400mg/kg, 800mg/kg dose) after administration were significantly decreased (p<0.01), and the levels of GSH-Px and SOD in the APS high-dose group (800mg/kg dose) were significantly increased (p<0.05, Fig. 3).

### Effect on FOXO3a, Wnt2, LRP5 and β-catenin mRNA gene expression in bone

**Figure 4:**
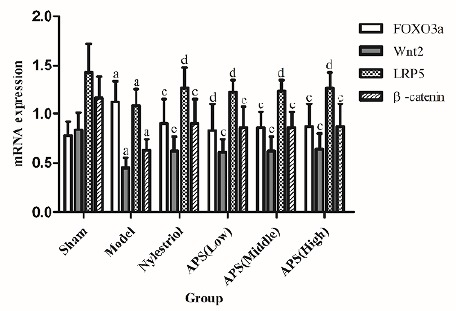
Effects on FOXO3a, Wnt2, LRP5 and β-catenin mRNA gene expression in bone. ^a^ P<0.01 compared with sham group; ^c^ P<0.01 compared with model group; ^d^ P<0.05 compared with model group.

 Compared with sham operation group, the gene expression of Wnt2, LRP5 and β-catenin mRNA in the bone tissues of the rats after bilateral ovarian resection were significantly decreased (p<0.01), but the expression level of FOXO3a mRNA was significantly increased (p<0.01). Compared with model group, the gene expression of Wnt2 and β-catenin mRNA in bone tissue of each dose group of APS rats were significantly increased (p<0.01), and the gene expression of LRP5 mRNA was also significantly increased (p<0.01), but the gene expression of FOXO3a mRNA of the middle- and high-dose APS groups (800mg/kg dose) was significantly increased (p<0.01, Fig. 4).

## Discussion

 The formation of PMOP is a long pathological process. Bone mineral density and bone pathological changes can reflect subtle changes of bone mass^11^. The bone metabolism index in serum can reflect state of bone metabolism, so it can be used as the main criterion for evaluating the therapeutic effect of drugs. Since the decrease in bone mechanical strength is an essential feature of PMOP, bone biomechanical properties are another reliable indicator for evaluating bone quality. Therefore, this experiment used bone mineral density, serum bone metabolism index, bone biomechanics and bone pathological changes as the main indicators.

 The maximum stress, maximum load and elastic modulus are important indicators of the biomechanical properties of bones. The determination of bone mineral density is helpful for direct evaluation of bone quality^12^. The experimental results show that APS can significantly increase the bone mineral density of the femur and increase the maximum stress, maximum load and elastic modulus of the ovariectomized rats, suggesting that the APS can increase the intrinsic strength of the bone and reduce the incidence of fracture.

 Estrogen is an upstream factor that regulates the Wnt/β-catenin pathway. Estrogen levels are reduced after menopause, and T cells are activated to promote osteoclast formation^13^. OC in serum can suggest an active state of newly formed osteoblasts, and ALP is an important biochemical indicator of bone formation and bone turnover. OC levels in serum can suggest an active state of newly formed osteoblasts, and ALP is an important biochemical indicator of bone formation and bone turnover. The experimental results showed that E_2_ in the serum of model group was significantly decreased, while the level of OC was significantly increased, suggesting that the high-transformation osteoporosis model was successfully established, which is consistent with the clinical characteristics of PMOP^14^. APS can significantly increase the levels of E_2_, ALP and BGP in the serum of rats, suggesting that APS has an estrogen-like effect and can prevent the formation of osteoporosis after ovariectomy by lowering the ALP and BGP values in the serum.

 The level of estrogen in the body of PMOP patients is continuously reduced, and the antioxidant capacity is gradually weakened. When the balance between the generation and elimination of reactive oxygen species is broken, the body produces oxidative stress. In this experiment, the oxidative stress state of PMOP was replicated by excising the bilateral ovaries of the rats. GSH-Px and SOD are important antioxidant enzymes in the body, which can eliminate the harmful substances produced by the body during metabolism, and can also reduce or eliminate the damage of superoxide anion radicals in the body and avoid cell damage caused by free radical accumulation. MDA is a degradation product of lipid peroxide, which can indicate the amount of oxygen free radicals and the degree of lipid peroxidation by detecting its concentration. The results of the experiment showed that APS significantly increased the levels of GSH-Px and SOD in the femoral tissue of ovariectomized rats and decreased the level of MDA, indicating that APS can improve the oxidative stress state of bone tissue in postmenopausal osteoporosis rats.

 More and more studies have shown that FoxOs transcription factor is a key factor affecting the redox balance of osteoblasts and the homeostasis of bones, and FoxOs-mediated oxidative stress plays an important role in the occurrence and development of osteoporosis^15^. FoxO3a is ubiquitous in bone and bone cells and has a high expression level. It stimulates the expression of functional genes such as free radical scavenging, and initiates transcriptional programs that regulate apoptosis, thereby constructing an oxidative stress line centered on FoxO3a^16^. The Wnt signaling pathway is an important intracellular signaling pathway involved in many basic life processes such as embryonic development and tissue and organogenesis. Inhibition of Wnt/LRP5/β-catenin signaling pathway leads to abnormal bone metabolism leading to osteoporosis, but activation of Wnt/LRP5/β-catenin promotes bone formation^17^ ^,^ ^18^. Under oxidative stress, FoxO is activated to competitively bind to β-catenin and translocate into the nucleus, resulting in the shift of limited β-catenin in the Wnt/β-catenin pathway from β-catenin/TCF-mediated transcription to FoxO-mediated transcription, thereby reducing Osteoblasts proliferate and differentiate and eventually form oxidative stress-induced osteoporosis^19^ ^,^ ^20^. Thus, inhibition of activation of the FoxO family members and activation of the Wnt /β-catenin pathway are essential for the prevention of oxidative stress-induced osteoporosis. The results of this experiment showed that APS can significantly increase the gene expression of Wnt2, β-catenin and LRP5 mRNA and inhibit the gene expression of FoxO3a mRNA. It can be seen that APS can effectively regulate FoxO3a / Wnt2 / β-catenin pathway and improve osteoporosis induced by oxidative stress in ovariectomized rats.

## Conclusion

 APS can effectively alleviate oxidative stress-mediated osteoporosis in ovariectomized rats, which may be related to its regulation of FoxO3a / Wnt2 / β-catenin pathway.
